# Real space electron delocalization, resonance, and aromaticity in chemistry

**DOI:** 10.1038/s41467-021-25091-8

**Published:** 2021-08-10

**Authors:** Leonard Reuter, Arne Lüchow

**Affiliations:** Institute of Physical Chemistry, RWTH Aachen University, Aachen, Germany

**Keywords:** Computational chemistry, Method development, Quantum chemistry

## Abstract

Chemists explaining a molecule’s stability and reactivity often refer to the concepts of delocalization, resonance, and aromaticity. Resonance is commonly discussed within valence bond theory as the stabilizing effect of mixing different Lewis structures. Yet, most computational chemists work with delocalized molecular orbitals, which are also usually employed to explain the concept of aromaticity, a ring delocalization in cyclic planar systems which abide certain number rules. However, all three concepts lack a real space definition, that is not reliant on orbitals or specific wave function expansions. Here, we outline a redefinition from first principles: delocalization means that likely electron arrangements are connected via paths of high probability density in the many-electron real space. In this picture, resonance is the consideration of additional electron arrangements, which offer alternative paths. Most notably, the famous 4*n* + 2 Hückel rule is generalized and derived from nothing but the antisymmetry of fermionic wave functions.

## Abstract (web summary)

The concept of delocalization, resonance and aromaticity are commonly discussed within electronic structure frameworks relying on specific wave function expansions. Here the authors propose a redefinition of these concepts from first-principles by investigating saddle points of the all-electron probability density.

## Introduction

Why do neutral atoms bind? In the early twentieth century, the covalent bond formulated by Lewis proposed a conundrum, which even leads Lewis to believe, that Coulomb’s law must fail at small distances.^1^ The first molecular application of Schrödingers wave equation^2^ by Heitler and London^3^ solved the puzzle mathematically but lacked a physically understandable explanation. Hellmann^4^ and later Ruedenberg^5^ related the covalent bond to the—essentially kinetic—“electron sharing”, whereas others—starting with Slater^6^—saw a predominately electrostatic origin. While the kinetic picture prevailed for molecules with first-row atoms (e.g., ${{{{{{{{{\rm{H}}}}}}}}}_{2}}^{+}$ and H_2_), it is disputed for larger systems^7–11^.

Pauling improved the Heitler–London picture of the chemical bond by giving even homoatomic bonds a certain ionic contribution, which gave rise to valence bond (VB) theory^12^. This mixing of ionic terms into the covalent wave function does not only lower the total energy—which is called resonance—, it also offers a coherent generalized picture of bonding, where covalent and ionic bonds are only the extreme forms of a continuously defined bond.

**Fig. 1:**
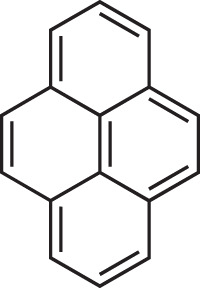
Pyrene. A planar aromatic system with 4*n π* electrons.

The stability of conjugated or aromatic systems is commonly attributed to the delocalization of *π* electrons, which is closely related to Ruedenberg’s “electron sharing”. Hückel had an elegant orbital-based explanation for the at first sight bizarre difference between aromatic and antiaromatic systems, i.e., with 4*n* + 2 and 4*n π* electrons respectively^13^. This apparent simplicity—among other things—eventually lead to the rise of molecular orbital (MO) theory. The Hückel rules’ seeming failure for some extended aromatic molecules like pyrene (Fig. 1)—which is not a failure of the Hückel method—is elegantly resolved with the concept of “conjugated circuits”^14–17^: the total aromaticity is ascribed to the existence of cyclic electronic subsystems, which abide the 4*n* + 2 rule. Furthermore, Baird showed, that the aforementioned Hückel rule is inverted for triplet systems, i.e., compounds with 4*n π* electrons are aromatic^18^.

Already before Hückel’s work, the diamagnetic properties of aromatic systems were attributed to electronic “ring currents”^19–22^. Theoretical studies used this notion of ring currents to explain aromaticity also in absence of a magnetic field and discussed the role of the ionic structures^23–26^. Notably, Shurki et al. stated that the flow of the electrons around the C_4_H_4_ ring is, therefore, interrupted, which is close to the line of arguments we intend to develop in this article.

Bader and coworkers developed the “delocalization index” (DI) as a real space measure of delocalization^27,28^ in the framework of the quantum theory of atoms in molecules (QTAIM)^29,30^. It is based on the one- and two-electron densities *ρ*(**r**) and ${\rho }_{2}({{{{{{{\bf{r}}}}}}}},{{{{{{{{\bf{r}}}}}}}}}^{\prime})$. Poater et al.^31^ subsequently introduced the mean of all DIs of *para*-related carbon atoms in a given six-membered ring (PDI) as a measure of aromaticity. However, as Martín Pendás and Francisco and the authors of this work showed recently, the DI is better understood as a measure of ionicity^32,33^. A more recent and very promising real-space approach to understand aromaticity is the wave function tiling by Schmidt and coworkers^34,35^.

In the present work, an extension to probability density analysis (PDA)^33,36,37^ is described. PDA was introduced as the all-electron equivalent of QTAIM and recovers Lewis structures by analyzing the local maxima of ∣Ψ∣^2^, which we label structure critical points (SCPs). Here, we add the saddle points, labeled delocalization critical points (DCPs), to the analysis. A DCP is the lowest point on the maximum probability path (MPP) between two adjacent SCPs, much like the lowest point on a mountain ridge. This mathematical framework allows for the probabilistic quantification of Ruedenberg’s “electron sharing”, which is increased freedom of movement (i.e., delocalization) lowering the kinetic energy—cf. increasing the box length for a particle in a box.

## Results

### Electron sharing in ${{{{{{{{{\rm{H}}}}}}}}}_{2}}^{+}$ and H_2_

**Fig. 2:**
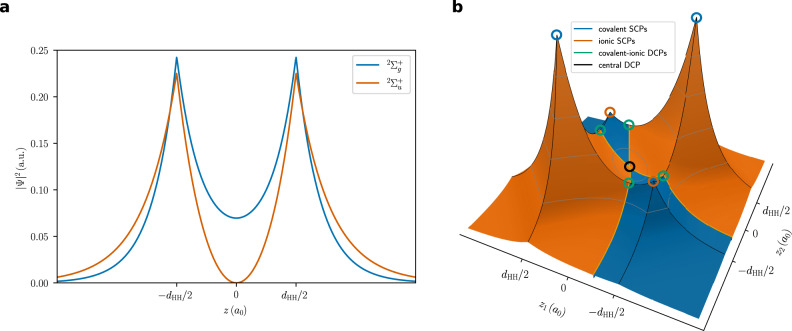
∣Ψ∣^2^ with the electrons on the bond axis *z*. **a**${{{{{{{{{\rm{H}}}}}}}}}_{{2}}^{+}}$ at the equilibrium distance (*d*_HH_ = 2.00 *a*_0_^40^). **b** H_2_ at the equilibrium distance (*d*_HH_ = 1.40 *a*_0_).

For ${{{{{{{{{\rm{H}}}}}}}}}_{2}}^{+}$, the two SCPs are simply the two proton positions (PDA and QTAIM are identical for one-electron systems). For infinitely separated protons, ∣Ψ∣^2^ at the DCP approaches zero, while it is non-zero for the bound state ${}^{2}{{{\Sigma }}}_{g}^{+}$ at equilibrium distance (Fig. 2). For the first excited state ${}^{2}{{{\Sigma }}}_{u}^{+}$, ∣Ψ∣^2^ at the DCP equals zero (node) independent of the proton–proton distance. For the infinitely dissociated system as well as for the ${}^{2}{{{\Sigma }}}_{u}^{+}$ state, the electron is thus not shared, since—while mathematically being at both protons—the electron could never move from one nucleus to the other. This impossibility of crossing a node of ∣Ψ∣^2^ is well established in the context of potential barriers, where an infinitely large barrier prevents particles from tunneling. In order to discuss barriers instead of low probability density, we define a probabilistic potential Φ, which is monotonically decreasing with ∣Ψ∣^2^. It is the scalar potential of the drift velocity **u**, which appears in the diffusion Monte Carlo method^38^ as well as in stochastic quantum mechanics^39^.

1 $${{\Phi }}=-\frac{\hslash }{2{m}_{e}}{{{{{{\mathrm{ln}}}}}}}\,| {{\Psi }}{| }^{2},\qquad {{{{{{{\bf{u}}}}}}}}=-\nabla {{\Phi }}=\frac{\hslash }{{m}_{e}}\frac{\nabla {{\Psi }}}{{{\Psi }}}$$

A probabilistic barrier between two SCPs can thus be defined as the value of Φ at the highest DCP on the connecting path (this barrier approaches infinity, if ∣Ψ∣^2^ at the DCP vanishes). This probabilistic barrier always coincides with an ordinary potential barrier—if there is one.

A VB wave function in the minimal basis of orbitals *φ*_K_ is chosen to investigate the two-electron bond in H_2_ (Eq. (2)).

2 $${{\Psi }}=N\left[(1-\eta ){\psi }_{{{{{{{{\rm{cov.}}}}}}}}}+\eta {\psi }_{{{{{{{{\rm{ion.}}}}}}}}}\right],\qquad {\varphi }_{{{{{{{{\rm{K}}}}}}}}}({{{{{{{\bf{r}}}}}}}})=\sqrt{\frac{{\zeta }^{3}}{\pi }}{e}^{-\zeta | {{{{{{{\bf{r}}}}}}}}-{{{{{{{{\bf{r}}}}}}}}}_{{{{{{{{\rm{K}}}}}}}}}| }$$

**Fig. 3:**
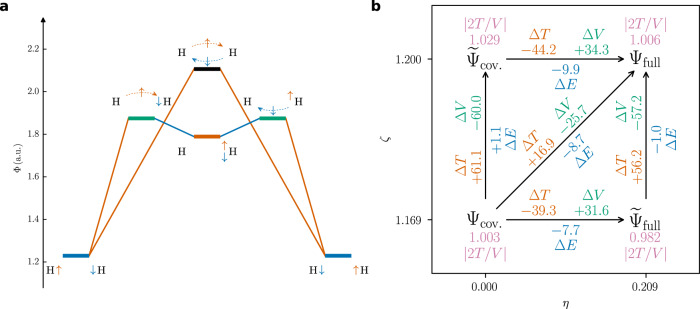
H_2_ at the equilibrium distance. **a** Probabilistic barriers for the concerted two-electron exchange and for the step-wise exchange with an ionic intermediate SCP. **b** Step-wise morphing of the covalent Heitler-London wave function Ψ(*η* = 0, *ζ* = 1.169) into the full VB wave function Ψ(*η* = 0.209, *ζ* = 1.200). All energies in m*E*_*h*_.

The coefficient *η* controls the ionic contribution and the exponent *ζ* the contraction of the wave function at the protons. Both are optimized in this ansatz, which is equivalent to CASSCF(2,2). Four SCPs and five delocalization critical points are identified with PDA. For all of these points, both electrons are positioned on the bond axis (Fig. 2b). The four SCPs are the covalent and ionic arrangements of the electrons. The central DCP is a second-order saddle point connecting the two covalent SCPs as well as the two ionic SCPs. It describes a concerted two-electron exchange. With a correlated wave function, the electrons would avoid each other, moving the central DCP away from the depicted plane. The four covalent-ionic DCPs describe the one-electron move from a covalent to an ionic arrangement or vice-versa. There are apparently two paths from one covalent SCP to the other: the concerted two-electron exchange via the central DCP and the step-wise electron exchange via two covalent-ionic DCPs and an ionic SCP. Since ∣Ψ∣^2^ is lower at the central DCP compared to the ionic-covalent DCP, the probabilistic barrier (i.e., the largest probabilistic potential Φ) is larger for the concerted two-electron exchange (Fig. 3a). The ionic arrangements thus serve a purpose which is analogous to a reaction intermediate. The probabilistic barrier is minimal for *η* = 0.42, which is close to the Hartree–Fock wave function (*η* = 1/2). Without optimization of the orbital exponent (i.e., *ζ* = 1), it is actually minimal exactly at *η* = 1/2. This is consistent with the established view, that “…the electrons in the HF-MO description of H_2_ are completely delocalized^[”26^.

In order to validate the proposed connection between resonance and the probabilistic barrier, the kinetic (*T*) and potential (*V*) contributions to the total stabilization have to be investigated. For H_2_, the resonance energy is defined as the energetic difference between the purely covalent (*η* = 0) Heitler–London wave function and the optimized (*η* = 0.21) VB wave function. Since the orbital coefficients *ζ* have been optimized, the virial ratio is close to one (∣2*T*/*V*∣ < 1.007) for both functions. It is not exactly equal to one, since the proton-proton distance has not been relaxed. Due to this non-relaxation, the virial ratio of the resonance energy is quite large: ∣2Δ*T*/Δ*V*∣ = 1.320. However, the energy difference is obviously still dominated by a decreasing potential and increased kinetic energy, qualitatively following the virial theorem. Yet, it can be shown—analogously to the “step-wise morphing” by Ruedenberg and coworkers^5,40^—that resonance in H_2_ is a kinetic stabilization.

Resonance can be divided into two “physical” steps: contraction of the wave function at the nuclei and the actual resonance (i.e., mixing in of the ionic contribution) (Fig. 3b). There are two ways of describing the step-wise morphing: mixing followed by contraction or contraction followed by mixing. We will proceed with the former since the contraction is destabilizing for the latter. The total energy difference of −8.7 m*E*_*h*_ can be attributed to 89% to the mixing, which, for its part, can be attributed to a kinetic stabilization (−39.3 m*E*_*h*_) outweighing an increase of the potential energy (+31.6 m*E*_*h*_). The remaining 11% resulting from orbital contraction lead—of course—to an overall increase of the kinetic energy. Yet, the nature of resonance remains essentially kinetic, justifying the discussion of the probabilistic barrier as the fundamental principle of resonance.

Going beyond singlet two-electron systems, the antisymmetry of fermionic wave functions has to be considered: the exchange of two same-spin electrons changes the sign of the wave function Ψ, while the probability density ∣Ψ∣^2^ remains the same, preserving the electrons indistinguishability. For a hypothetical triplet two-electron bond, having an antisymmetric spatial function, the picture thus changes drastically. There are only two SCPs (the covalent ones) and upon exchange of the two electrons, the sign of Ψ changes. Therefore, according to the intermediate value theorem, it is necessarily zero somewhere on any path connecting the two covalent SCPs, yielding an infinitely high probabilistic barrier. This is an easy explanation for why triplet H_2_ is not binding: the electrons are not shared. This is related to well-known energetic arguments: the introduction of a node raises the kinetic energy and therefore the total energy of a system.

### The two times 2*n* + 1 rule

Going beyond two electrons, the same arguments can still be used to predict and explain delocalization. For *n* same-spin electrons equally spaced on a ring, the rotation of the ring by 2*π*/*n* is described by the cyclic permutation *σ*_*n*_ = (12…*n*) ∈ *S*_*n*_. This permutation is positive for odd *n* and negative for even *n*: ${{{{{{{\rm{sgn}}}}}}}}({\sigma }_{n})={(-1)}^{n-1}$. If the sign of a permutation is negative, the sign of Ψ changes upon its application. If it is positive, Ψ is unchanged. Thus, again arguing with the intermediate value theorem, the rotation of an even-numbered ring of same-spin electrons is impossible, since it has to pass ∣Ψ∣^2^ = 0 (i.e., an infinitely large probabilistic barrier). This discussion of parity is related to the “electron-switch symmetry index” by Shurki et al.^26^ If the electrons were not confined to a ring, smaller cyclic permutations would still be possible (e.g., the permutation (123) for a system with 4 electrons). With the restriction to a ring, all of these smaller cycles also cross a node, since at least one electron has to pass another electron (e.g., the fourth in the above example).

**Fig. 4:**
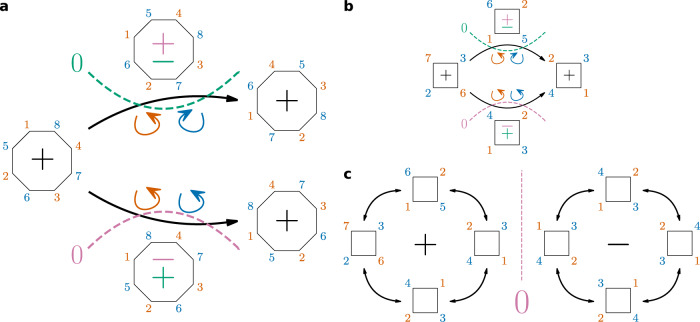
Antiaromatic systems. Comparison of counter-rotation and co-rotation of the spin-up (orange) and spin-down (blue) electrons for **a** an eight-electron system and **b** a four-electron system. The two potential nodes are depicted as dashed lines for both systems: purple (only counter-rotation is possible) and green (only co-rotation is possible). **c** Assuming a counter-rotation, a node separates two groups of spin-alternating covalent structures with identical individual cyclic orders of the spin rings (shown for the four-electron system).

If two rings (spin-up and spin-down) are combined (cf. the alternating spin structure of benzene), the intermediate value theorem apparently does not help anymore: rotating two rings of the same size at once always preserves the sign of Ψ independent of the parity. However, the rings can only rotate independently if both are odd-numbered. For these systems—which will be called aromatic—, the sign of the wave function depends only on the individual cyclic orders of the spin-up and spin-down rings. This means, that the sign is always constant as long as an electron does not ‘overtake’ a same-spin electron on the ring. If both rings are even-numbered—these systems will be called antiaromatic—, their rotation is restricted: they cannot rotate independently, since the rotation of only one ring would flip the sign. Furthermore, the combined rotation of two even-numbered rings is for any wave function restricted to be either a counter-rotation (the rings rotate in opposite direction) or a co-rotation (the rings rotate in the same direction): in between, these two combined rotations result in permutations of opposite signs, thus only one can be possible. This is demonstrated for an antiaromatic eight-electron system (Fig. 4a), where the two central intermediate arrangements have opposite signs due to an odd cyclic permutation of the spin-down electrons, while the two arrangements on the right-hand side are related to the left-hand arrangement by two cyclic permutations. The same demonstration can be done for an antiaromatic four-electron system (Fig. 4b, c).

Ultimately, the restricted rotation in antiaromatic systems always leads to the introduction of an additional node compared to aromatic systems, which is not related to same-spin electrons overtaking one another on the ring. This node, which splits all regions with identical individual cyclic orders of the spin rings in half, is comparable to the node in triplet H_2_.

The numbers of both, spin-up and spin-down electrons, therefore, have to be odd in order to have unrestricted circular delocalization: ${N}_{\uparrow },{N}_{\downarrow }\in \{2n+1:n\in {\mathbb{N}}\}$. This can be called the ‘two times 2*n* + 1’ rule. If the model of electrons on a ring is applied to the *π* system of singlet planar ring molecules (i.e., *N*_*↑*_ = *N*_*↓*_), the Hückel rule follows directly as 2(2*n* + 1) = 4*n* + 2. For *M*_*S*_ = ±1, which determines the symmetry of the triplet state, the Baird rule is retrieved: *N*_*↑*_ = *N*_*↓*_ ± 2 leads to the two equivalent rules 4*n* + 4 and 4*n*.

This real-space view of delocalization can also describe the aromaticity of polycyclic aromatic compounds like pyrene (Fig. 1), for which the Hückel rule is not valid. While the total number of *π* electrons is 16, there is still a plethora of possible rotations: e.g., on each six-membered ring (6 electrons), along with two six-membered rings (10 electrons), or including all electrons on the edges of the system (14 electrons). All these possible rotations lead to a stabilizing delocalization of the *π* electrons. This picture of polycyclic aromatic compounds is the real space analog of “conjugated circuits”^14–16^.

In addition, the concept of “paired electrons” is to some degree justified by and connected to the real space picture presented in this work: the electron pair is the smallest multi-electron system abiding the “two times 2*n* + 1” rule.

### MPPs in benzene and cyclobutadiene

While singlet cyclobutadiene (CBD) is considered to be antiaromatic, the D_6h_ symmetric benzene is aromatic according to the Hückel rule. With multi-reference *π*-only wave functions, the two alternating covalent spin structures are the most important (both by PDA weight^33^ and by the value of Φ) for the two systems in planar D_*n*h_ geometry (Supplementary Figs. 1 and 2). In order to explore delocalization, the MPPs connecting these major structures thus have to be investigated. The depicted paths, therefore, show the first halves of the rotations discussed in the previous paragraphs, while the non-depicted second halves are equivalent by symmetry. The most likely one-electron movements are identified by systematic screening of potential low-barrier paths.

**Fig. 5:**
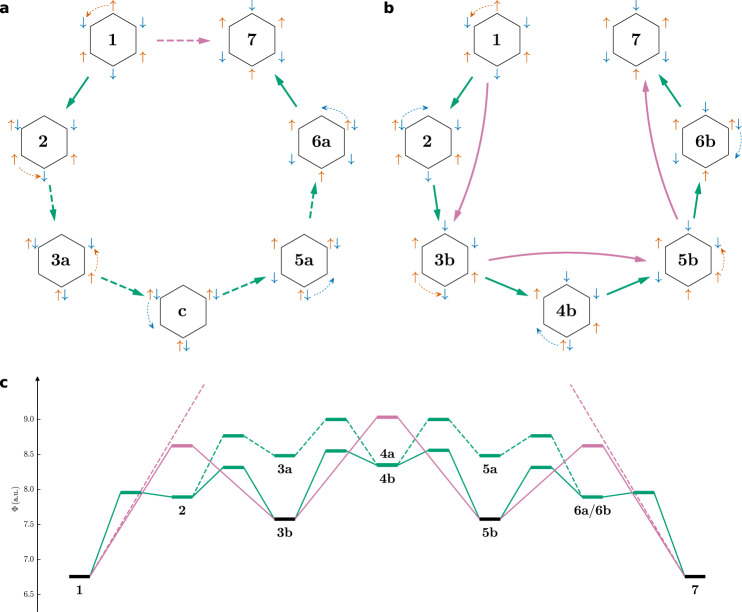
Maximum probability paths for the *π* system of benzene. Without restrictions (green) and restricted to covalent DCPs (purple). Spin-up and spin-down electrons rotate in **a** the same direction, **b** opposite directions. **c** Probabilistic barriers.

This search for major paths is somewhat analogous to work by Shurki et al.^26^ and seemingly related to the concept of diamagnetic ring currents^21,22^. However, like Maynau and Malrieu^25^ we do not restrict our search to paths, where all electrons move in the same circular direction (Fig. 5a). Instead, the spin-up and spin-down electrons can also rotate in opposite directions (Fig. 5b), which will eventually turn out to be more likely. Enforcing the picture of a ring current, the electrons most likely move between the covalent spin structures **1** and **7**, passing step-wise through the ionic SCPs **2**–**6a** without any concerted movement. A restriction of this same-direction path to covalent SCPs enforces a concerted movement of six electrons. Allowing the spin-up and spin-down electrons to rotate in opposite directions, the step-wise movement passes the two non-alternating covalent SCPs **3b** and **5b** in addition to the ionic SCPs **2**, **4b**, and **6b**. Here, a restriction to covalent spin structures only leads to concerted two-electron movements. All four investigated MPPs (same/opposite direction, restricted to covalent SCPs/no restriction) are compared regarding the probabilistic barrier (Fig. 5c).

In agreement with the previously discussed qualitative picture (two-times 2*n* + 1 rule), none of these paths crosses a node, i.e., there is no infinitely large barrier. In contrast to the prevalent picture of ring currents, the opposite direction paths are more likely, having a lower probabilistic barrier. Yet, it is easily imaginable, that an external magnetic field inverts this relation. These results contradict an earlier comparison^25^, which however only took concerted six-electron movements into account. The importance of ionic structures is in good agreement with the results by Shurki et al.^26^ for the same direction movement.

**Fig. 6:**
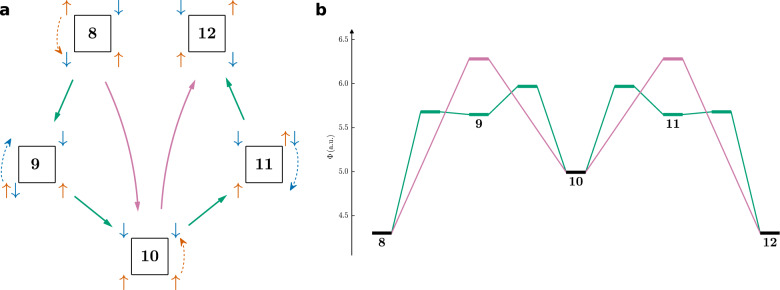
Maximum probability paths for the *π* system of singlet CBD. Without restrictions (green) and restricted to covalent DCPs (purple). **a** Spin-up and spin-down electrons rotate in opposite directions. **b** Probabilistic barriers.

For singlet CBD—again in agreement with the qualitative picture—the movement between the two major alternating spin structures is only found with the spin-up and spin-down electrons rotating in opposite directions, which is in perfect agreement with its paramagnetic properties. MPPs can be calculated like for benzene (Fig. 6).

**Fig. 7:**
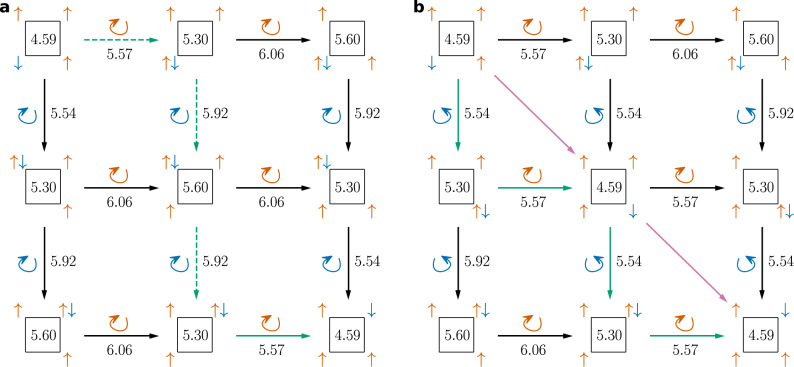
Maximum probability paths for the *π* system of triplet CBD. Comparison of all possible paths with probabilistic potentials for SCPs and DCPs. Resulting in MPPs without restrictions (green) and restricted to covalent DCPs (purple). Spin-up and spin-down electrons rotate in **a** the same direction, **b** opposite directions.

For the D_4h_ symmetric triplet CBD (which is aromatic according to the Baird rule) the opposite direction movement is again preferred over the same direction movement with both being possible. For this system, all screened paths can be depicted (Fig. 7).

## Discussion

To conclude, the freedom of movement for electrons is restricted by borders of low or even zero all-electron probability density ∣Ψ∣^2^. For planar circular systems, due to antisymmetry, the spin-up and spin-down electrons can only move independently if both are odd-numbered as additional nodes appear for even numbers. This leads directly to the Hückel and Baird rules of aromaticity (4*n* + 2 and 4*n* respectively) as well as to the electron pair. For H_2_ and the *π* systems in benzene and cyclobutadiene, the ionic structures act as an intermediate analog and play an important role in the broad phenomenon of delocalization. A Ruedenberg analysis (i.e., step-wise morphing) has shown, that the connection between the saddle points of ∣Ψ∣^2^ and bonding can explain the nature of resonance: the mixing of ionic contributions is essentially a kinetic stabilization. The in-depth analysis of aromaticity in benzene and triplet cyclobutadiene revealed that the counter-rotation of spin-up and spin-down electrons is more important than the same direction movement in absence of a magnetic field.

## Methods

### Hydrogen molecules

The equilibrium distances for ${{{{{{{{{\rm{H}}}}}}}}}_{2}}^{+}$ and H_2_ are taken from Ruedenberg and Schmidt^40^ and Huber and Herzberg^41^, respectively. The wave functions for the two states (${}^{2}{{{\Sigma }}}_{g}^{+}$ and ${}^{2}{{{\Sigma }}}_{g}^{-}$) of ${{{{{{{{{\rm{H}}}}}}}}}_{2}}^{+}$ is built from the 1s orbitals of Eq. (2)

3 $${{{\Psi }}}_{{}^{2}{{{\Sigma }}}_{g}^{\pm }}({{{{{{{\bf{r}}}}}}}})=\frac{1}{\sqrt{2\pm 2S}}\left[{\varphi }_{{{{{{{{\rm{A}}}}}}}}}({{{{{{{\bf{r}}}}}}}})\pm {\varphi }_{{{{{{{{\rm{B}}}}}}}}}({{{{{{{\bf{r}}}}}}}})\right],\qquad S=\langle {\varphi }_{{{{{{{{\rm{A}}}}}}}}}| {\varphi }_{{{{{{{{\rm{B}}}}}}}}}\rangle$$

The covalent and ionic VB structures of H_2_ are defined as

4 $${\psi }_{{{{{{{{\rm{cov.}}}}}}}}}({{{{{{{{\bf{r}}}}}}}}}_{1},{{{{{{{{\bf{r}}}}}}}}}_{2}) =\frac{1}{\sqrt{2+2{S}^{2}}}\left[{\varphi }_{{{{{{{{\rm{A}}}}}}}}}({{{{{{{{\bf{r}}}}}}}}}_{1}){\varphi }_{{{{{{{{\rm{B}}}}}}}}}({{{{{{{{\bf{r}}}}}}}}}_{2})+{\varphi }_{{{{{{{{\rm{B}}}}}}}}}({{{{{{{{\bf{r}}}}}}}}}_{1}){\varphi }_{{{{{{{{\rm{A}}}}}}}}}({{{{{{{{\bf{r}}}}}}}}}_{2})\right],\\ {\psi }_{{{{{{{{\rm{ion.}}}}}}}}}({{{{{{{{\bf{r}}}}}}}}}_{1},{{{{{{{{\bf{r}}}}}}}}}_{2}) =\frac{1}{\sqrt{2+2{S}^{2}}}\left[{\varphi }_{{{{{{{{\rm{A}}}}}}}}}({{{{{{{{\bf{r}}}}}}}}}_{1}){\varphi }_{{{{{{{{\rm{A}}}}}}}}}({{{{{{{{\bf{r}}}}}}}}}_{2})+{\varphi }_{{{{{{{{\rm{B}}}}}}}}}({{{{{{{{\bf{r}}}}}}}}}_{1}){\varphi }_{{{{{{{{\rm{B}}}}}}}}}({{{{{{{{\bf{r}}}}}}}}}_{2})\right].$$

The VB calculations were performed with XMVB^42,43^, all integrals were computed with Gaussian16^44^.

### Benzene and cyclobutadiene

Complete active space self-consistent field (CASSCF) geometry optimizations with an active space of all *π* orbitals have been performed with Molpro^45^ for benzene and triplet CBD. Each function of the Slater-type TZPae^46^ basis has been expanded into 14 primitive Gaussian-type functions^47,48^.

The investigated multi-reference, *π*-only wave functions have been obtained from the CASSCF expansion: the determinants are reduced to the *π* orbitals, while spin coupling and configuration interaction coefficients are left untouched. This way, the *π* system is optimized in the full presence of the *σ* and core electrons, which is more accurate than any effective *π* Hamiltonian. Note, that there is no need to calculate energies from these *π*-only wave functions while performing PDA.

With these wave functions, the reflection of any electron position through the molecule’s plane flips the sign but does not change ∣Ψ∣^2^ or Φ. The signs of the electrons’ *z* coordinates (if the molecule lies in the *xy* plane) is thus irrelevant for the presented results.

### Probability density analysis

The PDA was performed with the quantum Monte Carlo code Amolqc^49^. The spin is treated without approximation by arbitrary assignment of spin quantum numbers *m*_*s*_ = ±1/2 to electron indices, constrained bythe total quantum number *M*_*S*_. Evaluation of the spin functions (with *α*(*m*_*s*_) = 1/2 + *m*_*s*_ and *β* (*m*_*s*_) = 1/2 − *m*_*s*_) and sorting of the indices then leads to block diagonal Slater matrices. The determinants of these matrices are products of spin-up and spin-down determinants. This is well established in the quantum Monte Carlo community^38^.

The saddle points of ∣Ψ∣^2^ (DCPs) were identified by applying the Newton method to Φ. The maxima of ∣Ψ∣^2^ (SCPs) were identified with the steepest descent and L-BFGS^50^ methods. The PDA weights of equivalent structures have been summed with inPsights^51^.

In order to identify the MPPs for benzene and CBD, some paths are excluded heuristically due to their expected high barrier (e.g., paths with several electrons crossing the ring). All other paths are calculated and the one with the lowest maximal potential is identified as MPP (Fig. 7). If there are multiple paths with the same maximal potential, the path always follows the lowest saddle points (Fig. 7b). Analogously, all viable paths have been compared for benzene and singlet CBD (Supplementary Tables 5–7, Supplementary Fig. 3).

## Supplementary information

Supplementary Information

## Data Availability

The authors declare that all data supporting the findings of this study are available within the paper and its Supplementary Information files.

## Code availability

The algorithms used for the findings are described in the Methods section. The codes Amolqc and inPsights are available as open-source programs^49,51^. Any script used for evaluating data, starting other programs, or making figures is available upon request.

## References

[1] Lewis GN (1916). The atom and the molecule. J. Am. Chem. Soc..

[2] Schrödinger E (1926). Quantisierung als Eigenwertproblem. [Quantization as an eigenvalue problem]. Ann. Phys..

[3] Heitler W, London F (1927). Wechselwirkung neutraler Atome und homöopolare Bindung nach der Quantenmechanik. [Interaction of neutral atoms and homopolar bond according to quantum mechanics]. Z. Phys..

[4] Hellmann H (1933). Zur Rolle der kinetischen Elektronenenergie für die zwischenatomaren Kräfte. [On the role of the kinetic electron energy for the interatomic forces]. Z. Phys..

[5] Ruedenberg K (1962). The physical nature of the chemical bond. Rev. Mod. Phys..

[6] Slater JC (1933). The virial and molecular structure. J. Chem. Phys..

[7] Schmidt MW, Ivanic J, Ruedenberg K (2014). Covalent bonds are created by the drive of electron waves to lower their kinetic energy through expansion. J. Chem. Phys..

[8] Bacskay GB, Nordholm S, Ruedenberg K (2018). The virial theorem and covalent bonding. J. Phys. Chem. A.

[9] Nordholm S, Bacskay GB (2020). The basics of covalent bonding in terms of energy and dynamics. Molecules.

[10] Levine DS, Head-Gordon M (2017). Quantifying the role of orbital contraction in chemical bonding. J. Phys. Chem. Lett..

[11] Levine DS, Head-Gordon M (2020). Clarifying the quantum mechanical origin of the covalent chemical bond. Nat. Commun..

[12] Pauling L (1931). The nature of the chemical bond. Applications of results obtained from the quantum mechanics and from a theory of paramagnetic susceptibility to the structure of molecules. J. Am. Chem. Soc..

[13] Hückel E (1931). Quantentheoretische Beiträge zum Benzolproblem. I. Die Elektronenkonfiguration des Benzols und verwandter Verbindungen. [Quantum theoretical contributions to the benzene problem. I. The electron configuration of benzene and related compounds]. Z. Phys..

[14] Herndon WC (1973). Resonance energies of aromatic hydrocarbons. A quantitative test of resonance theory. J. Am. Chem. Soc..

[15] Swinborne-Sheldrake R, Herndon WC, Gutman I (1975). Kekulé structures and resonance energies of benzenoid hydrocarbons. Tetrahedron Lett..

[16] Randić M (1976). Conjugated circuits and resonance energies of benzoid hydrocarbons. Chem. Phys. Lett..

[17] Rickhaus M (2020). Global aromaticity at the nanoscale. Nat. Chem..

[18] Baird NC (1972). Quantum organic photochemistry. II. Resonance and aromaticity in the lowest ^8^*π**π*^*^ state of cyclic hydrocarbons. J. Am. Chem. Soc..

[19] Raman CV, Krishnan KS (1927). Magnetic double-refraction in liquids. Part I. Benzene and its derivatives. Proc. R. Soc. Lond. A.

[20] Lonsdale K (1937). Diamagnetic and paramagnetic anisotropy of crystals. Rep. Prog. Phys..

[21] Elvidge, J. A. & Jackman, L. M. Studies of aromaticity by nuclear magnetic resonance spectroscopy. Part I. 2-Pyridones and related systems. *J. Chem. Soc.* **859**, 859–866 (1961).

[22] Gomes JANF, Mallion RB (2001). Aromaticity and ring currents. Chem. Rev..

[23] Brooks H (1940). Diamagnetic anisotropy and electronic structure of aromatic molecules. J. Chem. Phys..

[24] McWeeny R (1953). The diamagnetic anisotropy of large aromatic systems V: interpretation of the results. Proc. Phys. Soc. A.

[25] Maynau D, Malrieu JP (1982). A valence bond effective Hamiltonian for the neutral states of *π* systems. 2. Results. J. Am. Chem. Soc..

[26] Shurki A, Hiberty PC, Dijkstra F, Shaik S (2003). Aromaticity and antiaromaticity: what role do ionic configurations play in delocalization and induction of magnetic properties?. J. Phys. Org. Chem..

[27] Bader RFW, Streitwieser A, Neuhaus A, Laidig KE, Speers P (1996). Electron delocalization and the Fermi hole. J. Am. Chem. Soc..

[28] Fradera X, Austen MA, Bader RFW (1999). The Lewis model and beyond. J. Phys. Chem. A.

[29] Bader RFW, Beddall PM (1972). Virial field relationship for molecular charge distributions and the spatial partitioning of molecular properties. J. Chem. Phys..

[30] Bader, R. F. W. & Nguyen-Dang, T. Quantum theory of atoms in molecules-Dalton revisited. in *Advances in Quantum Chemistry*, 14 edn. 63–124 (Academic Press, Inc., 1981).

[31] Poater J, Fradera X, Duran M, Solà M (2003). The delocalization index as an electronic aromaticity criterion: application to a series of planar polycyclic aromatic hydrocarbons. Chemistry.

[32] Martín Pendás A, Francisco E (2018). Decoding real space bonding descriptors in valence bond language. Phys. Chem. Chem. Phys..

[33] Reuter L, Lüchow A (2020). On the connection between probability density analysis, QTAIM, and VB theory. Phys. Chem. Chem. Phys..

[34] Liu Y, Frankcombe TJ, Schmidt TW (2016). Chemical bonding motifs from a tiling of the many-electron wavefunction. Phys. Chem. Chem. Phys..

[35] Liu Y, Kilby P, Frankcombe TJ, Schmidt TW (2020). The electronic structure of benzene from a tiling of the correlated 126-dimensional wavefunction. Nat. Commun..

[36] Lüchow A (2014). Maxima of ∣Ψ∣^2^: a connection between quantum mechanics and Lewis structures. J. Comput. Chem..

[37] Heuer MA, Reuter L, Lüchow A (2021). Ab initio dot structures beyond the Lewis picture. Molecules.

[38] Reynolds PJ, Ceperley DM, Alder BJ, Lester WA (1982). Fixed-node quantum Monte Carlo for molecules. J. Chem. Phys..

[39] Nelson E (1966). Derivation of the Schrödinger equation from Newtonian mechanics. Phys. Rev..

[40] Ruedenberg K, Schmidt MW (2007). Why does electron sharing lead to covalent bonding? A variational analysis. J. Comput. Chem..

[41] Huber KP, Herzberg G (1979). Molecular Spectra and Molecular Structure.

[42] Song L, Mo Y, Zhang Q, Wu W (2005). XMVB: a program for ab initio nonorthogonal valence bond computations. J. Comput. Chem..

[43] Chen Z (2015). XMVB 2.0: a new version of Xiamen valence bond program. Int. J. Quantum Chem..

[44] Frisch, M. J. et al. *Gaussian 16 Revision C.01*, https://www.gaussian.com (2016).

[45] Werner, H.-J. et al. *MOLPRO, Version 2019.2, a Package of Ab Initio Programs*, https://www.molpro.net (2019).

[46] Van Lenthe E, Baerends EJ (2003). Optimized Slater-type basis sets for the elements 1-118. J. Comput. Chem..

[47] O-ohata K, Taketa H, Huzinaga S (1966). Gaussian expansions of atomic orbitals. J. Phys. Soc. Jpn..

[48] Petersson GA, Zhong S, Montgomery JA, Frisch MJ (2003). On the optimization of Gaussian basis sets. J. Chem. Phys..

[49] Lüchow, A. et al. *Amolqc (v7.1.0). Zenodo*10.5281/zenodo.4562745 (2021).

[50] Nocedal J (1980). Updating quasi-Newton matrices with limited storage. Math. Comput..

[51] Heuer, M. A. & Reuter, L. *inPsights (v0.6.3). Zenodo.*10.5281/zenodo.4719297 (2021).

