# Supplementary material for: Real space electron delocalization, resonance, and aromaticity in chemistry
Source: Nat Commun. 2021 Aug 10;12:4820. doi: 10.1038/s41467-021-25091-8 (PMC8355119; doi:10.1038/s41467-021-25091-8)
Supplement: Supplementary file 1 — Supplementary Information [file 41467_2021_25091_MOESM1_ESM.pdf]

Real space electron delocalization, resonance, and aromaticity in chemistry

Leonard Reuter[1] & Arne Lüchow[1*]

[1] Institute of Physical Chemistry, RWTH Aachen University, Landoltweg 2, 52074 Aachen, Germany

[*] luechow@rwth-aachen.de

Supplementary Information

Supplementary Note 1

The normalization factor of the H_2 VB wave function is given in Equation 1:

$$N = \sqrt{\frac{S^2 + 1}{\left[1 - 2\eta\left(1 - \eta\right)\right]\left(S^2 + 1\right) + 4\eta\left(1 - \eta\right)S}} \tag{1}$$

Supplementary Figures

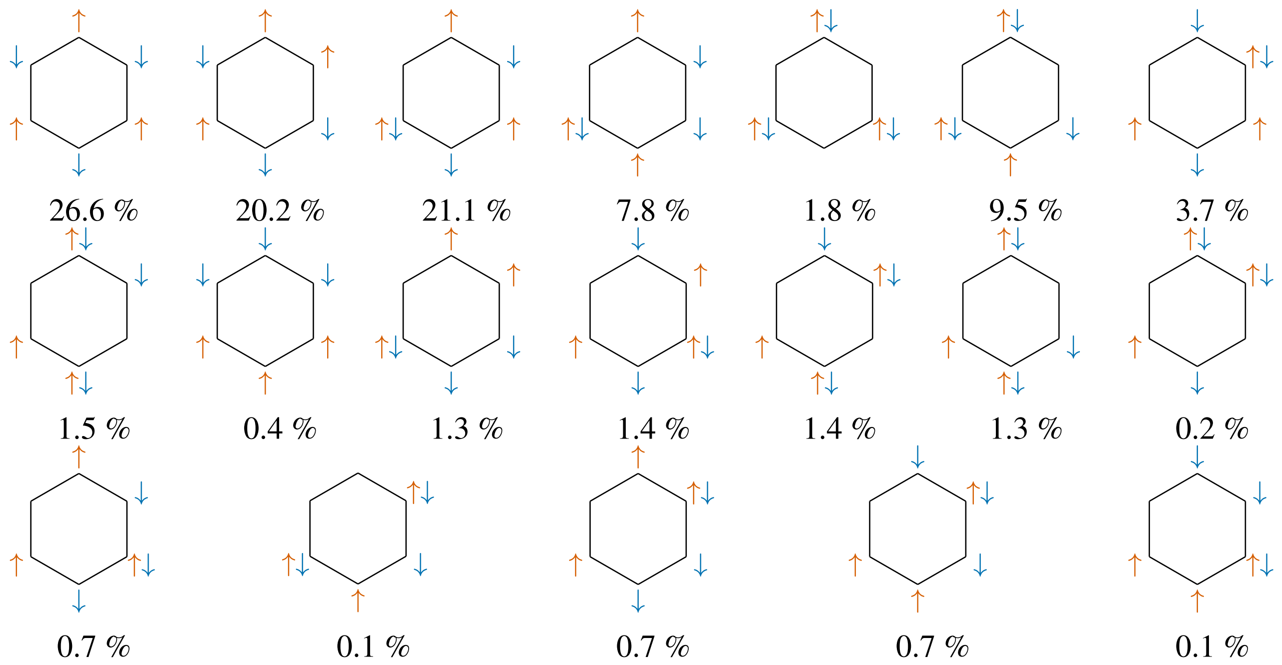

Supplementary Fig. 1: **Most important sets of spin structures for benzene with PDA weights.** For each set, only one of the equivalent spin structures is depicted. The spin structures are sorted by value of $|\Psi|^2$.

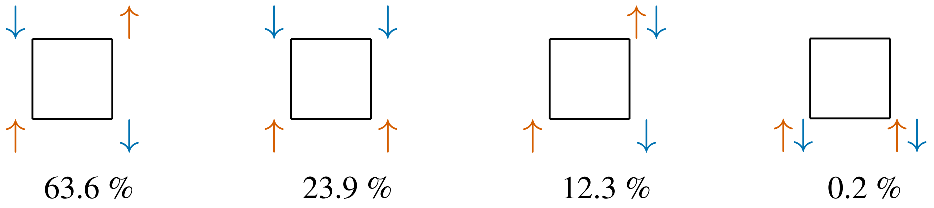

Supplementary Fig. 2: **Most important sets of spin structures for singlet CBD with PDA weights.** For each set, only one of the equivalent spin structures is depicted. The spin structures are sorted by value of $|\Psi|^2$.

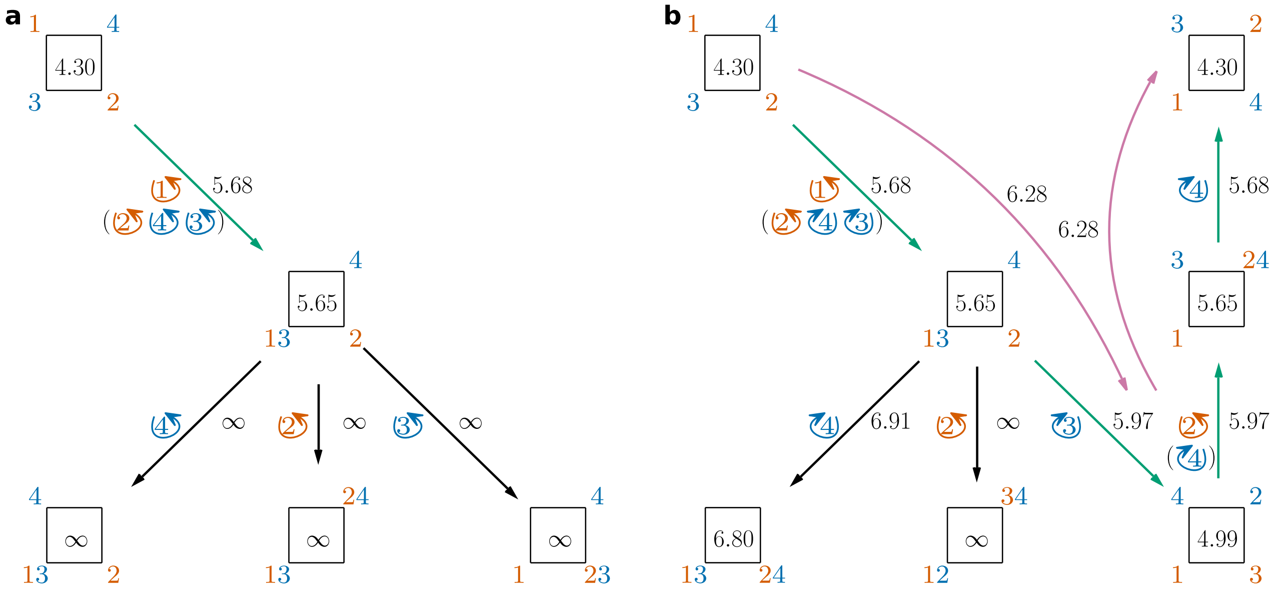

Supplementary Fig. 3: **Maximum probability paths for the π system of singlet CBD.** Comparison of possible paths for rotation in **a** the same direction, **b** opposite directions with probabilistic potentials. Spin-up electrons in orange, spin-down electrons in blue. Alternative equivalent paths in brackets.

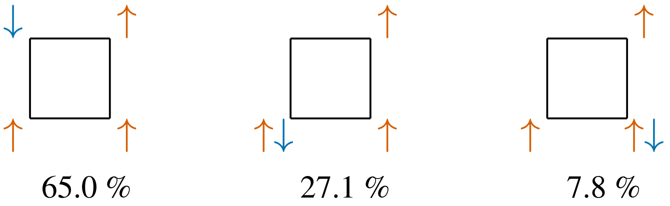

Supplementary Fig. 4: **Most important sets of spin structures for triplet CBD with PDA weights.** For each set, only one of the equivalent spin structures is depicted. The spin structures are sorted by value of $|\Psi|^2$.

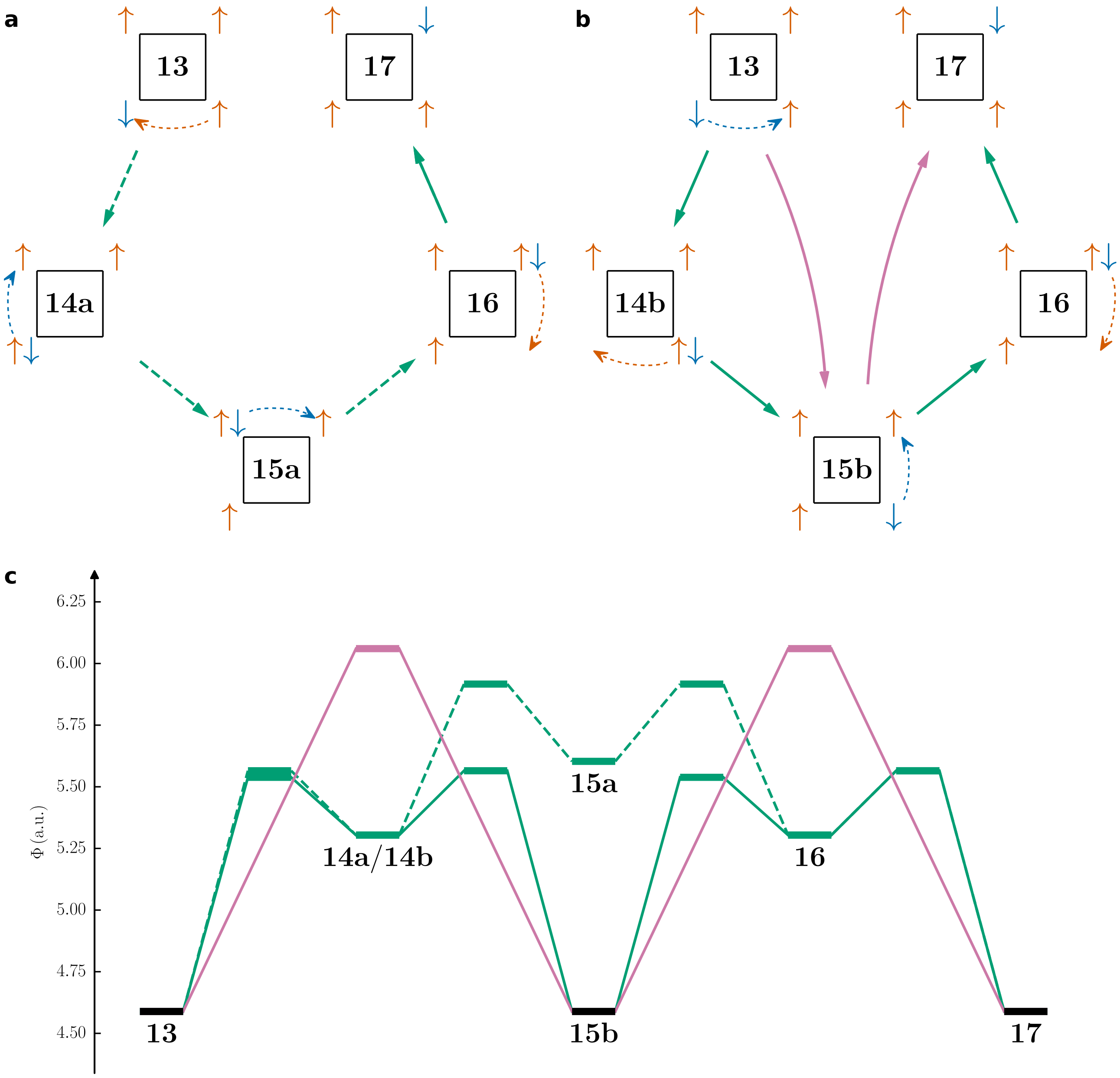

Supplementary Fig. 5: **Maximum probability paths for the π system of triplet CBD.** Without restrictions (green) and restricted to covalent DCPs (purple). Spin-up and spin-down electrons rotate in **a** the same direction, **b** opposite directions. **c** Probabilistic barriers.

Supplementary Tables

Supplementary Table 1: **Probabilistic potential for the critical points of the hydrogen molecule.** The covalent-ionic DCPs are found with the off-core coordinate $z = \pm 0.502\, d_{\mathrm{HH}}$.

critical point	cov. SCP	ion. SCP	cov.-ion. DCP	centr. DCP
Φ(a.u.)	1.229	1.791	1.876	2.108

Supplementary Table 2: **Energies for different wave functions of the hydrogen molecule.** The wave functions are described in the article.

wave function	$E(E_h)$	$T(E_h)$	$V(E_h)$
$\Psi_{\mathrm{cov.}}$	-1.13905382	1.14531687	-2.28437069
$\tilde{\Psi}_{\mathrm{cov.}}$	-1.13793069	1.20642681	-2.34435750
Ψ_{full}	-1.14778742	1.16226263	-2.31005005
$\tilde{\Psi}_{\mathrm{full.}}$	-1.14678675	1.10602725	-2.25281401

Supplementary Table 3: **Energies and geometries for benzene and cyclobutadiene.** All values are calculated with CASSCF/TZPae.

System	State	Active space	Symmetry	Energy (E_h)	d_{CC}(Å)	d_{CH}(Å)
C_6H_6	$^1A_{1g}$	(6, 6)	D_{6h}	-230.84423492	1.393	1.074
C_4H_4	$^3A_{2g}$	(4, 4)	D_{4h}	-153.72860396	1.437	1.069
C_4H_4	$^1B_{1g}$	(4, 4)	D_{4h}	-153.74520550	1.442	1.069

Supplementary Table 4: **Probabilistic potential for the critical points of benzene.** Saddle points are denoted with '**-**'.

critical point	**1, 7**	**2, 6a, 6b**	**3a, 5a**	**3b, 5b**	**4a**	**4b**	**1-2, 6a-7, 6b-7**	**1-3b, 5b-7**
Φ(a.u.)	6.756	7.891	8.482	7.576	8.352	8.344	7.955	8.623

**1-7**	**2-3a, 5a-6a**	**2-3b, 5b-6b**	**3a-4a, 4a-5a**	**3b-4b**	**4b-5b**	**3b-5b**
13.644	8.765	8.315	8.998	8.550	8.558	9.031

Supplementary Table 5: **Comparison of different paths for benzene for the same direction movement.** Potentials of possible DCPs for all intermediates. Spin-up electrons (1 to 3) in orange, spin-down electrons (5 to 6) in blue. Starting configuration is the spin-alternating covalent structure 1-4-2-5-3-6 (counter-clockwise). DCPs of the MPP (and equivalent paths) in green. Highest DCP of the MPP in bold. DCPs which are higher than that highest DCP in purple.

	1	2	3	4	5	6
**1**	7.95	7.95	7.95	7.95	7.95	7.95
**2**		8.77	8.78	8.90	8.96	8.89
**3a**			**9.00**	9.29	9.33	9.31
**4a**				**9.00**	**9.00**	**9.00**
**5a**					8.77	8.78
**6a**						7.95

Supplementary Table 6: **Comparison of different paths for benzene for the opposite direction movement.** Potentials of possible DCPs for all intermediates. Spin-up electrons (1 to 3) in orange, spin-down electrons (5 to 6) in blue. Starting configuration is the spin-alternating covalent structure 1-4-2-5-3-6 (counter-clockwise). DCPs of the MPP (and equivalent paths) in green. Highest DCP of the MPP in bold. DCPs which are higher than that highest DCP in purple.

	1	4	2	5	3	6
**1**	7.95	7.95	7.95	7.95	7.95	7.95
**2**		8.31	8.78	9.22	8.77	9.28
**3b**			8.55	8.91	8.91	8.55
**4b**				**8.56**	8.99	9.39
**5b**					8.31	8.31
**6b**						7.95

Supplementary Table 7: **Alternative paths for benzene, that cannot be discarded in Table 5**. The DCPs of these paths are written in black in Table 5. DCPs of the MPP (and equivalent paths) in green. DCPs which are higher than that highest DCP in purple.

	1	2	3	4	5	6
**3a′**		9.00		9.33	9.31	9.29
**3a″**		9.36	9.42		9.40	9.37
**3a‴**		9.48	9.49	9.49		9.48
**3a⁗**		9.40	9.37	9.36	9.42	
**5a′**					7.95	

Supplementary Table 8: **Probabilistic potential for the critical points of singlet CBD.** Saddle points are denoted with '-'.

crit. pt.	**8, 12**	**9, 11**	**10**	**8-8,11-12**	**9-10,10-11**	**8-10,10-12**
Φ(a.u.)	4.301	5.649	4.993	5.679	5.969	6.281

Supplementary Table 9: **Probabilistic potential for the critical points of triplet CBD.** Saddle points are denoted with '-'.

crit. pt.	**13, 15b, 17**	**14a, 14b, 16**	**15a**	**13-14b,15b-16**	**13-14a,14b-15b,16-17**
Φ(a.u.)	4.589	5.304	5.603	5.538	5.565

**14a-15a,15a-16**	**13-15b,15b-17**
5.917	6.060
